# Biotechnology-enhanced immunoassay for accurate determination of HT-2 toxin in edible insect samples

**DOI:** 10.1007/s00604-025-07146-5

**Published:** 2025-04-16

**Authors:** Raúl Sancho-García, Fernando Navarro-Villoslada, Fernando Pradanas-González, Henri O. Arola, Bettina Glahn-Martínez, Tarja K. Nevanen, Elena Benito-Peña

**Affiliations:** 1 Department of Analytical Chemistry, Faculty of Chemistry, Universidad Complutense de Madrid, Plaza de las Ciencias, Ciudad Universitaria, Madrid, 28040 Spain; 2 VTT Technical Research Centre of Finland Ltd, Tekniikantie 21, Espoo, 02044 Finland; 3 Centre for Military Medicine, Finnish Defence Forces, Tukholmankatu 8 A, Helsinki, 00301 Finland

**Keywords:** Non-competitive immunoassay, HT-2 toxin, Food safety, Immune complex, Edible insects, Cricket flour

## Abstract

Consumption of edible insects is common in non-Western countries of Africa, Asia, Oceania, and Latin America. However, their consumption has significantly increased in Europe in recent years thanks to their remarkable nutritional properties. Edible insects provide a valuable source of high-quality proteins, fats, minerals, and vitamins. Nevertheless, the absence of global regulatory guidelines poses a risk associated with their consumption due to the potential presence of pathogens and contaminants, such as mycotoxins, which are toxic compounds produced by fungi and represent a major threat to food and feed safety. Our approach integrates advanced nanobiotechnology to develop fluorescent antibodies by conjugating a recombinant superfolder green fluorescent protein (sfGFP) with a single-chain antibody (scFv). This fusion allows for precise detection of the immune complex formed between the HT-2 toxin and a biotinylated anti-HT-2 antibody. Additionally, we employed advanced computational tools, including AlphaFold and MOE, to deepen our understanding of the binding interactions present in the immune complex, confirming the strong interaction between the Fab/HT-2 toxin immunocomplex and the scFv antibody fragment, in contrast to the weaker binding observed with the Fab/T-2 toxin and the scFv. The method demonstrates high sensitivity, with an EC_50_ of 10.3 ± 0.6 ng mL^−1^, a dynamic range of 3.4 ± 0.1 to 31 ± 3 ng mL^−1^, a limit of detection of 0.43 ng mL^−1^, and a limit of quantification of 1.2 ng mL^−1^ in buffer solution. The assay exhibited excellent precision, with a reproducibility of 4% and no cross-reactivity with other mycotoxins. Application to contaminated cricket flour yielded recoveries between 91 and 133%, with coefficients of variation from 6 to 13%. These results indicate that the developed immunoassay is highly sensitive, selective, and reliable for detecting HT-2 toxin in food matrices, providing a promising tool for mycotoxin screening in food safety.

**Graphical Abstract:**

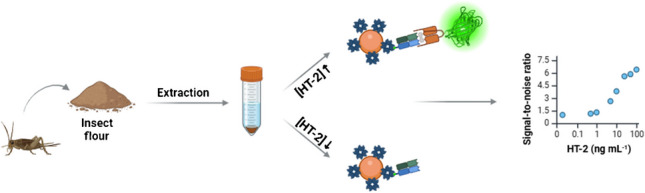

**Supplementary Information:**

The online version contains supplementary material available at 10.1007/s00604-025-07146-5.

## Introduction

The rising consumption of insect-derived products marks a shift toward sustainable and innovative food sources. These foods are increasingly seen as a solution to global issues like malnutrition and the environmental impact of traditional livestock farming. Insect-based foods are rich in proteins, essential minerals, and vitamins [1], making them a valuable nutritional addition. Furthermore, insect farming is highly resource-efficient, requiring less feed, water, and land while emitting fewer greenhouse gases [2]. However, challenges remain, particularly cultural resistance in Western societies and food safety concerns, such as contamination by toxic substances like mycotoxins. While the European Union has approved certain insect-based foods [3–6], comprehensive safety standards for contaminants are still under development.

Mycotoxins, toxic secondary metabolites produced by filamentous fungi, can contaminate food and pose significant health risks. The Food and Agriculture Organization (FAO) estimates that up to 25% of food crops globally are affected by mycotoxins, although some reports suggest even higher figures [7]. Environmental factors such as temperature and humidity are critical for fungal growth and mycotoxin production, highlighting the need for effective detection systems. One particularly concerning mycotoxin is the HT-2 toxin [8], a secondary metabolite produced by *Fusarium* fungi during the degradation of its precursor, T-2 toxin. HT-2 interferes with protein and DNA synthesis and induces apoptosis [9], posing significant health risks. This toxin is commonly found in cereals and their derivatives [10] and is more frequently detected than T-2 due to its higher stability and persistence in food products. To address the associated health risks, the European Union has established regulatory limits for HT-2 and T-2 toxins in cereals [11]. However, extending these regulations to include insect-based products is essential to safeguard public health and build consumer trust in this emerging food category.

Traditional detection methods including high-performance liquid chromatography (HPLC) and mass spectrometry (MS) [12] are highly sensitive but impractical for routine use due to their cost and complexity. Immunoassays have emerged as a more feasible alternative for mycotoxin detection [13–16]. Nevertheless, conventional competitive immunoassays face challenges such as structural changes in labeled toxins that can reduce antibody affinity, highlighting the need for more reliable and cost-effective detection methods.

The novelty of this research lies in the application of advanced biotechnological innovations to overcome these challenges. Specifically, we developed a fluorescence-based non-competitive immunoassay for the detection of HT-2 toxin, utilizing a unique pseudosandwich assay format. Unlike traditional competitive assays, this method enhances sensitivity and specificity, particularly for small molecules like HT-2 toxin, by forming an immune complex (IC) that can be detected without altering the mycotoxin’s structure.

**Fig. 1:**
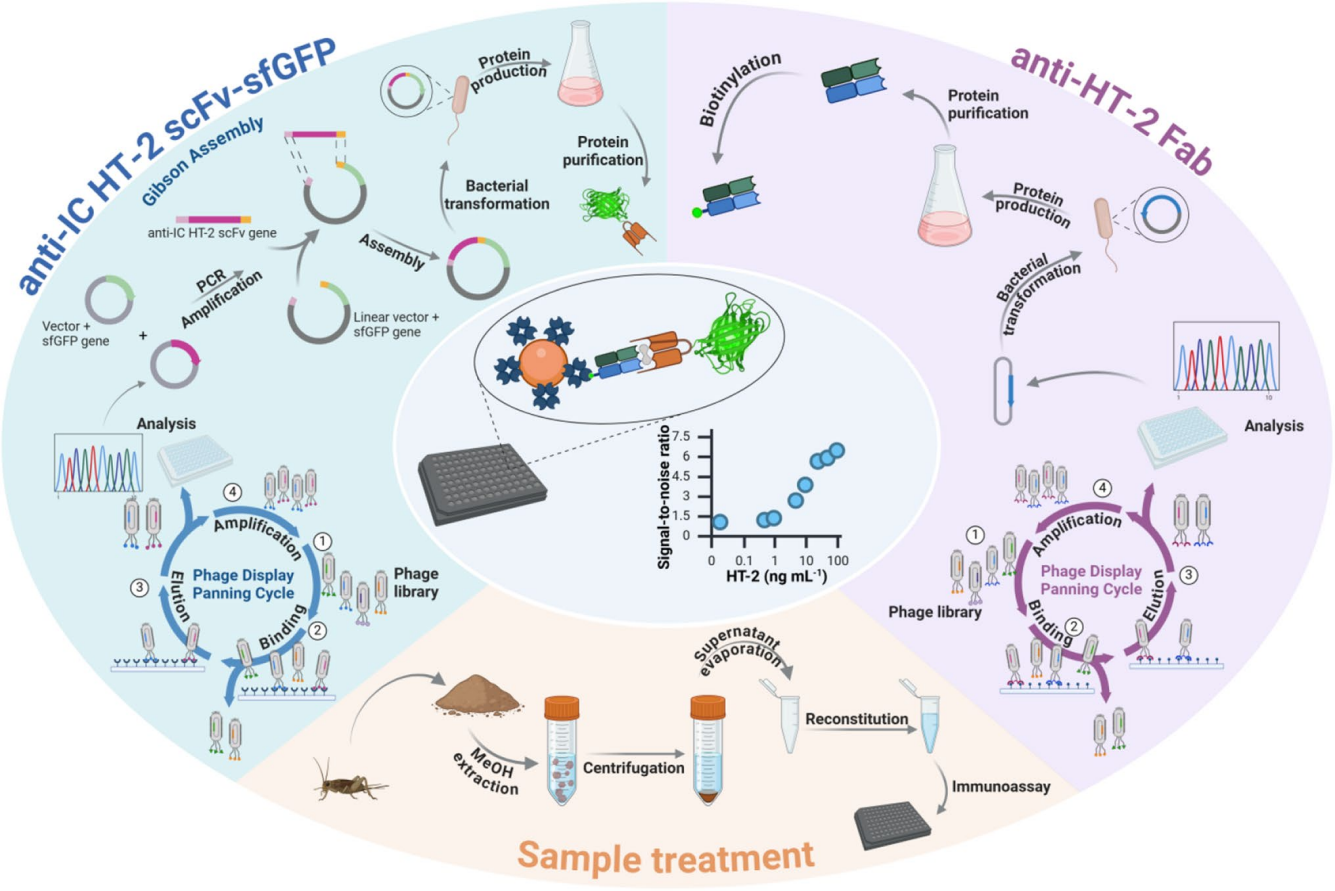
Schematic representation of the non-competitive fluorescence immunoassay for the detection of HT-2, the procedures followed to obtain the immunoreagents and the sample treatment. Quantifying the mycotoxin is performed using a non-competitive assay, in which a selective anti-HT-2 Fab immobilized in magnetic beads recognizes the HT-2 toxin and, subsequently, an anti-IC HT-2 scFv-sfGFP interacts with the anti-HT-2 Fab − HT-2 immune complex. Figure created in BioRender

A key feature of our approach is the fusion of a superfolder green fluorescent protein (sfGFP) with a single-chain variable fragment (scFv) antibody. This recombinant fusion protein binds specifically to the IC formed between the HT-2 toxin and a biotinylated anti-HT-2 Fab fragment, which is supported on magnetic particles (Fig. 1). The use of sfGFP ensures a stable and robust fluorescent signal, while the scFv fragment enhances the specificity and binding affinity of the assay [20–22]. This method eliminates the need of labelling secondary antibodies with reporter molecules for detection [17].

In this context, the innovative application of bioinspired recombinant antibodies, specifically the sfGFP-scFv fusion, when combined with recombinant Fab fragment, presents numerous advantages. This approach significantly enhances assay reproducibility across batches, ensures specificity, and provides a reliable long-term supply of recombinant antibodies [18, 19]. Furthermore, the application of computational modeling has provided us with a deeper understanding of antibody-antigen interactions, immune complex stability estimation, and an overall overview of the assay mechanism. Indeed, this strategy may open doors for bioanalytical researchers to optimize assay performance and redesign more effective immunoassays, paving the way for enhanced sensitivity and specificity robustness.

This work represents a significant advancement in the field of mycotoxin detection, offering a novel, highly sensitive, and specific method for monitoring HT-2 toxin contamination in edible insect products. By applying this assay to samples of cricket flour, we demonstrate its practical applicability in ensuring food safety in the expanding insect-based food industry. This methodology not only meets the regulatory standards for mycotoxin levels but also promotes consumer confidence in the safety of these novel foods.

The development of this fluorescence-based non-competitive immunoassay for HT-2 toxin detection exemplifies the integration of cutting-edge biotechnological tools in food safety. It sets a new standard for mycotoxin monitoring in insect-based products, ensuring that this sustainable food source can meet both regulatory and consumer expectations. This work paves the way for the broader adoption of innovative immunoassays in food safety applications, reinforcing the role of biotechnology in addressing global food security challenges.

## Experimental section

### Reagents and consumables

HT-2 toxin, T-2 toxin and fumonisin B1 (FB1) were purchased from Fermentek Ltd (Jerusalem, Israel). Deoxynivalenol (DON) and zearalenone (ZEA), kanamycin, chloramphenicol, imidazole, isopropyl β-D- 1-thiogalactopyranoside (IPTG), *E. coli* BL21 (DE3) pLysS, and PBS (0.01 M phosphate-buffered saline, 0.138 M NaCl, 2.7 mM KCl, pH 7.4) were supplied by Sigma-Aldrich (St. Louis, MO, USA). Antibody fragment (anti-HT-2 (10) Fab) and a sequence coding for single-chain variable antibody fragment (anti-IC HT-2 (10) scFv) were from Patent EP3129403 owned by VTT Technical Research Centre (Espoo, Finland). QIAquick PCR clean-up kit and QIAprep spin mini-prep kit were from Qiagen (Hilden, Germany). NEBuilder HiFi DNA Assembly Master Mix, competent *E*. *coli* NEB 5α were from New England biolabs (Ipswich, MA, USA). Phusion Hot Start II HF DNA polymerase, EZ-link sulfo NHS-LC-LC-Biotin, Pierce™ Protease Inhibitor Tablets, PageRuler™ Prestained Protein Ladder (10 to 180 kDa), Pierce™ Protein Free (PFBB) blocking buffer, and 96 black flat-bottom well plates were obtained from Thermo Scientific (Waltham, MA, USA). Bacterial cell lysis buffer and Luria Broth (LB) medium were obtained from NZYTech (Lisbon, Portugal). HisTrap FF crude, PD-10, and illustra NAP 5 columns were purchased from GE Healthcare (Chicago, IL, USA). SpeedBeads magnetic neutravidin-coated particles were from Cytiva (Marlborough, MA, USA). All primers and plasmid containing the gene encoding for anti-IC HT-2 (10) scFv were bought from Integrated DNA Technologies (Coralville, IA, USA). pET41-sfGFP plasmid was kindly donated by Dr. Barderas (ISCIII, Spain). Black cricket (*Gryllus bimaculatus Sp*) powder was purchased from JR Unique Foods Ltd. (Udon Thani, Thailand) and processed as received.

Ultrapure water obtained from a Millipore Milli-Q water purification system was used throughout.

Stock solutions of the mycotoxins at a 1 mg mL^−1^ concentration in DMSO stored at 4 °C were used to prepare standard solutions daily by dilution in assay buffer.

### Instrumentation

UV−vis absorption spectra were measured with a Varian Cary 3-Bio spectrophotometer, and steady-state fluorescence measurements were carried out on a Horiba Fluoromax-4 TCSPC spectrofluorometer equipped with a 150-W xenon lamp. The fluorescence quantum yield of the anti-IC-HT-2 scFv-sfGFP fusion protein was determined in phosphate buffer pH 7.4 using fluorescein as standard (*Φ*_f_ = 0.89 ± 0.04 in NaOH) [20], with excitation at 450 nm. All measurements were carried out in triplicate, and absorption was always kept below 0.1 at the absorption maximum.

Microplate fluorescence measurements were made using a CLARIOstar reader from BMG Labtech. The instrument was operated, and the data was processed using MARS, the manufacturer’s original software. The excitation wavelength was (470 ± 15) nm, and detection was monitored at (513 ± 20) nm, using a matrix scan mode (10 × 10).

Solutions were centrifuged on a miniSpin microcentrifuge or a 5804R centrifuge from Eppendorf AG (Hamburg, Germany). Samples were evaporated on a Savant DNA SpeedVac 110 apparatus from Holbrook (NY, USA). The microplates used in the study were washed in a HydroFlex plate washer equipped with magnetic support from Tecan (Männedorf, Switzerland).

### Molecular cloning of the recombinant anti-IC-HT-2 scFv-sfGFP fusion protein

**Fig. 2:**
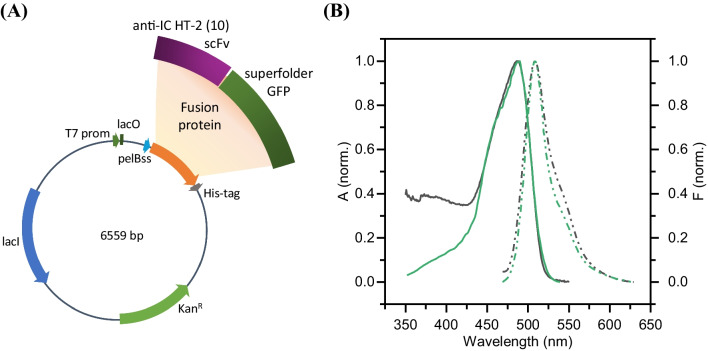
**A** Main features of the expression vector used to obtain a translational fusion protein consisting of anti-IC-HT-2 scFv and sfGFP. The vector pET41 includes a T7 promotor, the lac repressor (lacI) and lac operator (lacO) to suppress uninduced expression, kanamycin resistance (Kan.^R^), and a histidine affinity tag (HisTag). **B** Normalized absorption (solid line) and fluorescence spectra (dashed line) for sfGFP (green) and anti-IC-HT- 2 scFv-sfGFP (gray) in PBS (10 mM, pH 7.4)

Anti-IC-HT-2 scFv fused with sfGFP was constructed using molecular cloning using the Gibson assembly method. Briefly, the encoding genes were PCR-amplified from the commercial plasmids by using the Phusion Hot Start II DNA polymerase and the primer sets stated in Table S1, allowing the incorporation of a GGGS linker between the anti-IC-HT-2 scFv and sfGFP when using the NEBuilder HiFi DNA Assembly Master Mix for the assembly reaction. The pET41 plasmid containing the fusion of the sfGFP and the anti-IC-HT-2 scFv (Fig. 2A) was transformed into DH5*α E. coli* cells by heat shocking and plated on LB-agar containing kanamycin 50 µg mL^−1^. A single colony was grown overnight in 5 mL of a LB-kanamycin solution of the same concentration, and plasmids were extracted and purified using the QIAprep Spin Miniprep Kit. The correct assembly of the coding sequence of the constructs was verified using Sanger sequencing. Subsequently, the codifying plasmid was transformed into BL21 (DE3) pLysS *E. coli* chemically competent cells by heat shock and the resulting cells were plated and selected on LB-agar containing 50 µg mL^−1^ kanamycin and 33 µg mL^−1^ chloramphenicol.

### Protein expression and purification

A single colony was picked from the LB-agar plate and was grown overnight on a 15-mL LB preculture supplemented with 50 µg mL^−1^ kanamycin and 33 µg mL^−1^ chloramphenicol. An aliquot of the abovementioned preculture was transferred to a 200 mL LB-kanamycin-chloramphenicol culture and grown at 37 °C until an optical density at 600 nm (OD600) of 0.6–0.8 was reached. Then, 100 µL of IPTG (1 M) was added to the culture to induce the expression and grown for 16 h at the same temperature. After, the cells were harvested by centrifugation (5000 g for 10 min at 4 °C), and the pellet was frozen for at least 3 h at −80 °C, resuspended in a commercial lysing buffer with protease inhibitor cocktail and lysed by sonication on ice (VibraCell Ultrasonic Processor 130 W 20 kHz, Ampl 70%). The recombinant protein was purified from the cell lysate by a HisTrap affinity column, and a PD10 exclusion column was used to change the protein buffer to PBS. Finally, protein purity was evaluated by SDS-PAGE gel analysis (Figure S1), and concentrations were calculated by using theoretical extinction coefficients at 280 nm (ExPASy ProtParam tool). The purified fluorescent protein was stored at 4 °C protected from light.

### Antibody fragment (Fab) biotinylation

The anti-HT-2 Fab, obtained by phage display [21], was biotinylated following the manufacturer’s instructions. The anti-HT-2 Fab fragment was incubated with a 20-fold molar excess of activated biotin reagent for 30 min at room temperature. The excess biotin was efficiently removed with a molecular exclusion column (NAP-5 columns), ensuring the purity of the biotinylated anti-HT-2 Fab, which was eluted with PBS.

### Molecular docking

Molecular docking simulations were conducted to obtain the computational molecular modeling of anti-HT-2 Fab–HT-2 toxin complex against the single-chain fragment variable anti-IC HT-2 scFv-sfGFP. The 3D structure of the anti-HT-2 Fab and the anti-IC HT-2 scFv-sfGFP was predicted with the AlphaFold (AF) model (see Supporting Information). From the confidence metrics used by the AF model, both structures were predicted with an overall high accuracy. The DiffDock-L and Autodock Vina implementation in the Neurosnap platform was used to predict the anti-HT-2 Fab–HT-2 and the anti HT-2 Fab–T-2 interactions. The top-ranked structure of the anti-HT-2 Fab predicted by AF model was used as the input structure, whereas the number of predictions produced for each mycotoxin was ten. The best-ranked anti-HT-2 Fab/mycotoxin complex was subsequently used in the molecular docking simulations with the anti-IC HT-2 scFv-sfGFP. Simulations were performed using the Molecular Operating Environment (MOE) from Chemical Computing Group (Montreal, Canada) [22]. All the structures were prepared for molecular docking calculations using the Structure Preparation and the 3D Protonation applications in MOE 2022.02 software. Due to the multitude of possible interactions between the anti HT-2 Fab and anti-IC HT-2 scFv-sfGFP structures, simulations were performed by using the mycotoxin (HT-2 or T-2) and the anti-HT-2 Fab complementarity-determining regions (CDRs) as the target receptor region, and the CDRs of the anti-HT-2 anti-IC HT-2 scFv-sfGFP as the ligand region. The rigid receptor with the Protein–Protein docking protocol was used for the molecular docking of the anti-HT-2 Fab/mycotoxin complex against anti-IC HT-2 scFv-sfGFP using default parameters. In MOE, receptor-ligand binding affinities with all possible binding geometries are prioritized based on a numerical value called *S-score*. The ligand position with the lowest *S-score* tends to establish a strong interaction with the receptor in a specific region [23]. On the other hand, the root mean square deviation between the pose before refinement and the pose after refinement (*rmsd_refine*) was also considered as a criterion to choose the most probable docking of the anti-HT-2 Fab-mycotoxin complex against anti-IC HT-2 scFv-sfGFP. PyMOL (from DeLano Scientific LLC, San Francisco, CA, USA) [24] was used to visualize the anti-HT-2 Fab/mycotoxin complexes, the molecular docking of the complex against the anti-IC HT-2 scFv-sfGFP as well as visualize both acceptor and donor hydrogen bonding interactions between the mycotoxins and both antibodies using the plugin *Show contacts* [25] for displaying polar contacts.

### Non-competitive fluorescence immunoassay

The immunoassay, performed in 96-well black plates, utilizes neutravidin-coated magnetic particles (NMBs) as solid support. These NMBs were previously functionalized with the biotinylated anti-HT-2 Fab by mixing 19.80 µL (10 mg mL⁻^1^) NMBs with 14.66 µL of the anti-HT- 2 Fab (450 µg mL⁻^1^) and 965 µL of assay buffer (PFBB). After a 30 min incubation with gentle agitation at room temperature, the antibody-conjugated particles (Fab-MBs) were washed three times with the washing buffer (PBST, consisting of PBS containing 0.05% Tween-20) and then resuspended in 264 µL of the PFBB assay buffer, a step that ensures the particles are in an optimal environment for the subsequent stages of the immunoassay.

The general assay protocol is initiated by blocking a black 96-well plate with 200 µL PFBB assay buffer for 1 h at room temperature with gentle shaking. The plate is then washed three times with a wash buffer. Then 10 µL of Fab-MBs solution, 30 µL of sample or calibration standard, and 30 µL of anti-IC-HT-2 scFv-sfGFP (55.6 µg mL^−1^) are added to each well. The plate is then incubated for 15 min at room temperature with shaking, followed by three washes with PBST. Finally, the magnetic particles are resuspended in 50 µL of PBS. Fluorescence was monitored with a CLARIOstar microplate reader, measuring the emitted fluorescence intensity at 513 nm with excitation at 470 nm. A schematic of the assay protocol is depicted in Fig. 1.

The fluorescence data obtained from the assay images in the presence (B) and in the absence of the analyte (B_0_) were normalized using the following expression:

$$Normalized\;response=\frac{\left(B-B_0\right)}{B_\infty-B_0}$$

where* B*_∞_ is the fluorescence obtained in the presence of an excess of HT-2. Experimental data were fitted to the following four-parameter sigmoidal logistic Eq. (4-PLC) by using the software Origin 2021:

$$Normalized\;signal=A_{min}+\frac{A_{max}-A_{min}}{1+\left(\frac{\left[HT-2\right]}{{EC}_{50}}\right)^b}$$

where *A*_*max*_ and *A*_*min*_ are the asymptotic maximum and minimum of the normalized signal, respectively, the *EC*_*50*_ is the HT-2 concentration at the inflection point, and *b* represents the slope of the curve at the inflection point, which are parameters that characterize the sensitivity of the method since they are related to the affinity of the antibody for the target compound. The dynamic range (DR) was calculated as the analyte concentrations leading to a normalized response between 20 and 80% [26], and the limit of detection (LOD) as the mean of the blank signal plus three times its standard deviation.

The cross-reactivity (CR) of other common mycotoxins produced by fungi of the genus *Fusarium* was estimated using the optimized non-competitive immunoassay. The cross-reactivity values were calculated according to the equation:

$$CR\;\left(\%\right)=\frac{{EC}_{50}^{HT-2}}{{EC}_{50}^{cross-reactant}}x100$$

### Analysis of insect flour samples

Black cricket flour samples were commercially obtained and analyzed by LC–MS/MS to exclude possible natural HT-2 contaminants [27]. Different concentrations of the target analyte were spiked following a similar protocol described in Peltomaa et al. [28]. Briefly, a concentrated toxin solution was distributed at different points throughout the sample, mechanically agitated using a vortex for uniformity, and allowed to equilibrate for 16 h at room temperature prior to analysis. The HT-2 toxin spiked in 1 g of sample was then subjected to an extraction process involving 5 mL of MeOH/water (70/30, v/v), shaken for 1 h at room temperature, and centrifugated at 6000 g for 10 min. Following this, 1 mL aliquots of the supernatant was evaporated to a volume of 100 µL using a SpeedVax 110 evaporator. The extract was then diluted 1:2 (initial volume) with PFBB, and the treated samples were subjected to fluorescence immunoassay.

## Results and discussion

### Design and characterization of recombinant anti-IC HT-2 scFv-sfGFP

The anti-IC-HT-2 scFv-sfGFP fusion protein was obtained using molecular biology techniques [29]. A glycine-serine linker separates the two entities, and a histidine tag at the C-terminal end facilitates protein purification. PCR-amplified genes were successfully fused in the pET41 vector using the Gibson assembly reaction, and anti-IC-HT-2 scFv-sfGFP was appropriately expressed as a soluble form in *E. coli* BL21 (DE3) pLysS.

Figure 2B shows the absorption and emission spectra for anti-IC-HT-2 scFv-sfGFP in PBS. The absorption and fluorescence peaks fell at 488 and 508 nm, respectively. The fluorescence quantum yield, determined against fluorescein as the standard (*Φ*_f_ = (0.89 ± 0.04) in NaOH) [20], was (0.30 ± 0.02) in PBS and the excitation wavelength *λ*_exc_ = 450 nm. All measurements were made in triplicate, and absorption at the excitation wavelength was always below 0.1. These results are consistent with the reported data for sfGFP, which exhibits an absorption peak at 488 nm, an emission peak at 510 nm, and a fluorescent quantum yield of 0.65 in aqueous solutions [30]. The fact that the shape and position of the fluorescence peaks remained unchanged indicates that fusion with the scFv protein did not alter the structure of sfGFP.

### Molecular docking simulations

#### Anti-HT-2 Fab–mycotoxin interaction

**Fig. 3:**
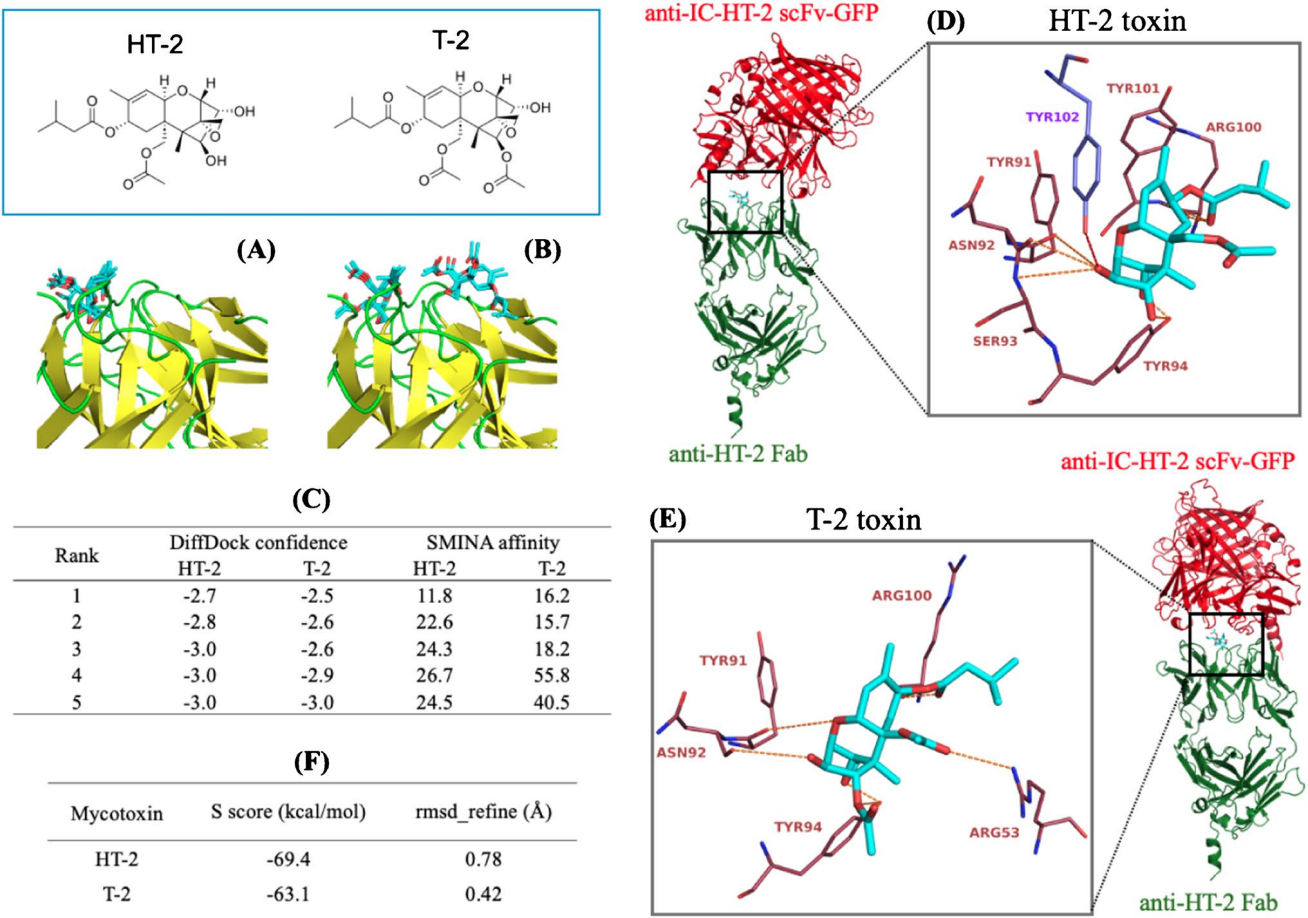
The five top-ranked DiffDock-based molecular dockings of anti-HT-2 Fab with **A** HT-2 and **B** T-2 toxins. **C** Confidence metrics from the DiffDock-L model of the HT-2 and T-2 docking with the anti-HT-2 Fab. Top-ranked MOE prediction of the anti-HT-2 Fab/mycotoxin complex interaction against anti-IC HT-2 scFv-sfGFP for **D** HT-2 and **E** T-2 toxins. **F** Docking scores of the anti-HT-2 Fab/mycotoxin complex interaction against anti-IC HT-2 scFv-sfGFP

The accuracy of the predicted dockings was assessed by the DiffDock confidence function and the SMINA affinity (Fig. 3C). The five top-ranked molecular dockings of HT-2 and T-2 against anti-HT-2 Fab, according to the DiffDock confidence function, are shown in Fig. 3A and B, respectively. Higher DiffDock scoring levels tend to mean better predictions, whereas lower affinity values are better, indicating stronger binding between the ligand and protein, which suggests a more favorable interaction. Generally speaking, a DiffDock prediction can be considered reasonably accurate if all or most of the predicted ligands are within very close proximity and orientation to one another. If many ligands are predicted to be around many different regions, it generally means the model is not as confident in the prediction. In the case of the HT-2 docking, 90% of the predicted ligands were around a single anti-HT-2 Fab region, whereas for the T-2 docking, the predicted ligands were around two different Fab regions with a 60:40 ratio, being one of the regions the same as for HT-2 toxin. The insets of Fig. 3D and E illustrate the acceptor and donor hydrogen bond interactions between the top-ranked molecular docking of the HT-2 and T-2 toxins with the anti-HT-2 Fab, with the next four top-ranked interactions available in the supplemental material, focusing on the amino acids located in the CDRs of the anti-HT-2 Fab. Both toxins seem to bind to the same region, mainly the CDR-3 pocket, including Tyr91, Asn92, Tyr94 of CDR-L3, and Arg101 of CDR-H3. The HT-2 toxin also seems to interact with the Tyr100 of CDR-H3, whereas the T-2 toxin interacts with Arg53 of CDR-H2. As observed, the HT-2 toxin exhibits distinct acceptor–donor hydrogen bond interactions compared to those observed with the T-2 toxin. Although these differences do not result in a substantial variation in the inhibition IC_50_ values obtained in competitive assays [31], they play a crucial role in defining the specific molecular interactions that occur between HT-2 and the scFv antibody fragment during the immunocomplex formation. The minor variation in the experimentally determined IC_50_ values may be attributed to the enhanced interaction of the amino acid Tyr94 with the hydroxyl group of HT-2. Conversely, its interaction with the T-2 toxin may be affected by steric hindrance presented by the carbonyl group, or by loading effects.

#### Anti-HT-2 Fab/mycotoxin complex against anti-IC HT-2 scFv-sfGFP

The top-ranked HT-2 and T-2 docking with the anti-HT-2 Fab predicted by DiffDock-L model was used as the input structure for the molecular dockings simulation with the anti-IC HT-2 scFv-sfGFP. The molecular dockings of anti-HT-2 Fab/HT-2 and anti-HT-2 Fab/T-2 complexes against anti-IC HT-2 scFv-sfGFP with the minimum *S-score* and the lowest *rmsd_refine* are shown in Fig. 3D and E, whereas Fig. 3F shows the docking scores of the selected interaction models. The anti-HT-2 Fab/HT-2 complex showed a minimum *S-score* of −69 kcal/mol, whereas the anti-HT-2 Fab/T-2 complex showed a minimum S-score of −63 kcal/mol. Thus, the anti-HT-2 Fab/HT-2 showed a stronger binding affinity than the anti-HT-2 Fab/T-2 complex against the anti-IC HT-2 scFv-sfGFP. The HT-2 mycotoxin seems to interact with the Tyr102 of the CDR-H3 pocket of the anti-IC HT-2 scFv-sfGFP fusion protein, whereas no amino acid of the anti-IC HT-2 scFv-sfGFP appears to interact with the T-2 mycotoxin. On the other hand, the interactions between both antibodies should also play an essential role in predicting the molecular docking of the anti-HT-2 Fab/mycotoxin complex against anti-IC HT-2 scFv-sfGFP.

### Bioassay optimization

**Fig. 4:**
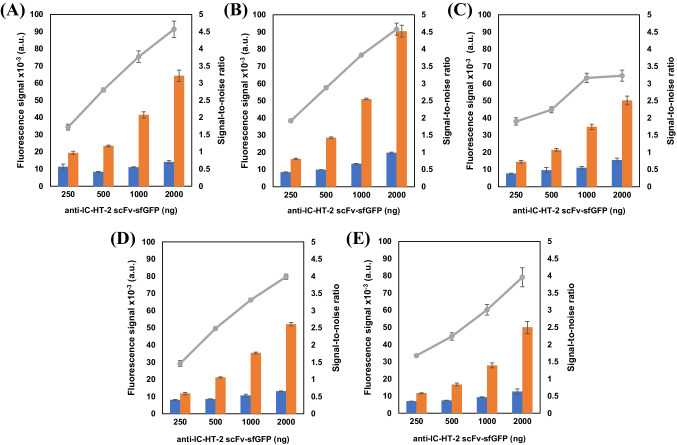
Fluorescence results obtained in the absence (orange bars) and presence of 10 ng mL^−^^1^ of HT-2 (blue bars), together with the signal-to-noise ratio (gray symbols), obtained by using different amounts of anti-IC-HT-2 scFv-sfGFP (0.25, 0.5, 1, and 2 µg) together with varying amounts of biotinylated anti-HT-2 Fab: **A** 150 ng, **B** 250 ng, **C** 500 ng, **D** 750 ng, and **E** 1000 ng per well. Constant concentration of NMBs of 5 ng/well. Results are shown as mean signals ± the standard error of the mean (*n* = 3)

Optimization of the bioassay method is necessary to determine HT-2 toxin with high sensitivity and in a simple, rapid, and reproducible way. First, different combinations of the amounts of biotinylated anti-HT-2 Fab (150 to 1000 ng) and anti-IC-HT-2 scFv-sfGFP (208 ng to 1667 ng) were used in the presence and the absence of 10 ng mL^−^^1^ of HT-2. As the results in Fig. 4 demonstrate, for the same concentration of anti-HT-2 Fab, the fluorescence intensity increases with the concentration of scFv-sfGFP, as does the signal-to-noise ratio, increasing the sensitivity. However, at higher anti-HT-2 Fab concentrations, the relative sensitivity decreases due to the saturation of the anti-HT-2 Fab binding sites. The best results were obtained with 1667 ng of anti-IC-HT-2 scFv-sfGFP (23.8 µg mL^−^^1^) and 250 ng of anti-HT-2 Fab (3.6 µg mL^−^^1^).

**Fig. 5:**
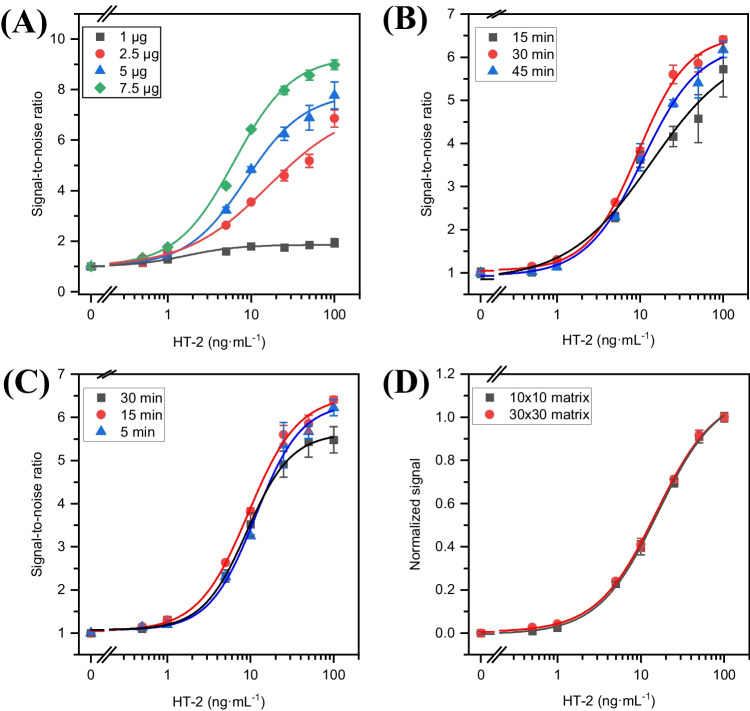
**A** Dose–response curves using different amounts of NMBs per well: 1 ng (black squares), 2.5 ng (red circles), 5 ng (blue triangle), and 7.5 ng (green diamond) in the presence of 1667 ng of anti-IC-HT-2 scFv-sfGFP and 250 ng of anti-HT-2 Fab. **B** Calibration curves obtained by varying the incubation times between NMB and biotinylated anti-HT-2 Fab. The second incubation was kept at 15 min, and optimized reagent concentrations were used. **C** Calibration curves obtained by varying the incubation times between Fab-MB, HT-2, and anti-IC-HT-2 scFv-sfGFP. The first incubation was held at 30 min, and optimized reagent concentrations were used. **D** Calibration plots for HT-2 obtained after measuring with a matrix of 10 × 10 (black squares) and 30 × 30 (red circles). Results are shown as mean signal ± standard error (*n* = 3)

From the optimized protein concentrations, four concentrations (1 ng to 7.5 ng/well) of NMBs were evaluated. As shown in Fig. 5A, the results underscore the significance of 7.5 ng/well of NMBs as the optimal amount of particles, providing the best sensitivity and reproducibility of the measurements. Higher particle concentrations were not tested, as high concentrations can cause particle aggregation, which can reduce the specificity of the assay and result in inefficient particle separation during washing, which can affect the accuracy of the results. In addition, it may increase costs without providing additional benefits.

Since incubation time usually has a direct effect on the analytical characteristics of immunoassays, different times were evaluated to achieve efficient binding of biotinylated anti-HT-2 Fab to magnetic particles with streptavidin (15, 30, and 45 min) and between the antibody-toxin immune complex (anti-HT-2 Fab/HT-2) with anti-IC-HT-2 scFv-sfGFP (5, 15, and 30 min). Figure 5B demonstrates that a first incubation of 30 min consistently leads to better analytical characteristics. The Holm-Bonferroni method (95% confidence level) was employed to compare the *EC*_*50*_, slope, and *A*_*max*_ parameters obtained from fitting the data to a 4-PLC to select the second incubation time (Fig. 5C). This method revealed no significant differences after 5 min of incubation. However, due to the higher reproducibility of the signal, an incubation time of 15 min was selected for further assays.

Furthermore, the dimensions of the measurement matrices employed in the surface scan were assessed, as this significantly impacts the measurement time. To this end, measurement matrices of 10 × 10 and 30 × 30 were used when concentrations in the range of 0 to 100 ng mL^−^^1^ of HT- 2 were assayed. As can be noted in Fig. 5D, there are no significant differences between the calibrations obtained with the evaluated matrices. Therefore, a 10 × 10 matrix was considered an optimal choice due to its potential for faster measurements.

### Analytical characterization

**Fig. 6:**
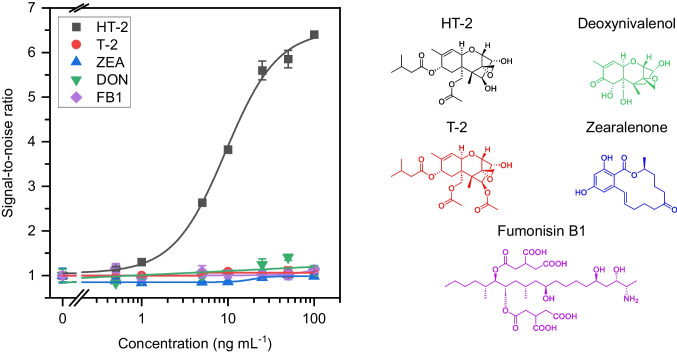
**A** Dose−response plots for HT-2 (black squares), T-2 (red circles), DON (green triangles), ZEA (blue triangles), and FB1 (purple diamonds) obtained by using Fab-MBs and scFv-sfGFP in PFBB. **B** Chemical structure of the target mycotoxins. Results are shown as mean signal ± standard error (*n* = 3)

Figure 6A shows the normalized calibration curve of the immunoassay obtained with HT-2 standards at concentrations ranging from 0 to 100 ng mL^−^^1^ and with previously optimized conditions. EC_50_ value was (10.3 ± 0.6) ng mL^−^^1^, and the LOD was determined to be 0.43 ng mL^−^^1^. The dynamic range, taken as the range of concentrations giving rise to a signal variation to the blank between 20 and 80%, was between 3.4 and 31 ng mL^−^^1^. The reproducibility of the bioassay was demonstrated by the relative standard deviation (RSD) of 4% for intra-day assays (*n* = 3). Compared to previously reported immunoassays for HT-2 quantification, the developed non-competitive immunoassay shows equal or superior performance in sensitivity and reproducibility (Table S4).

In order to implement the biosensor in the analysis of insect flour samples, its selectivity for HT-2 and other common mycotoxins produced by fungi of the genus *Fusarium*, which may be found together with the HT-2 toxin, were tested. The mycotoxins evaluated were HT-2 toxin, T-2 toxin, deoxynivalenol (DON), zearalenone (ZEA), and fumonisin B1 (FB1) [32]. As observed in Fig. 6A, all the possible interferent species studied showed a CR of less than 1%, including T-2, despite its high structural similarity to HT-2 toxin, confirming the high selectivity of the assay.

### Matrix effect evaluation

**Fig. 7:**
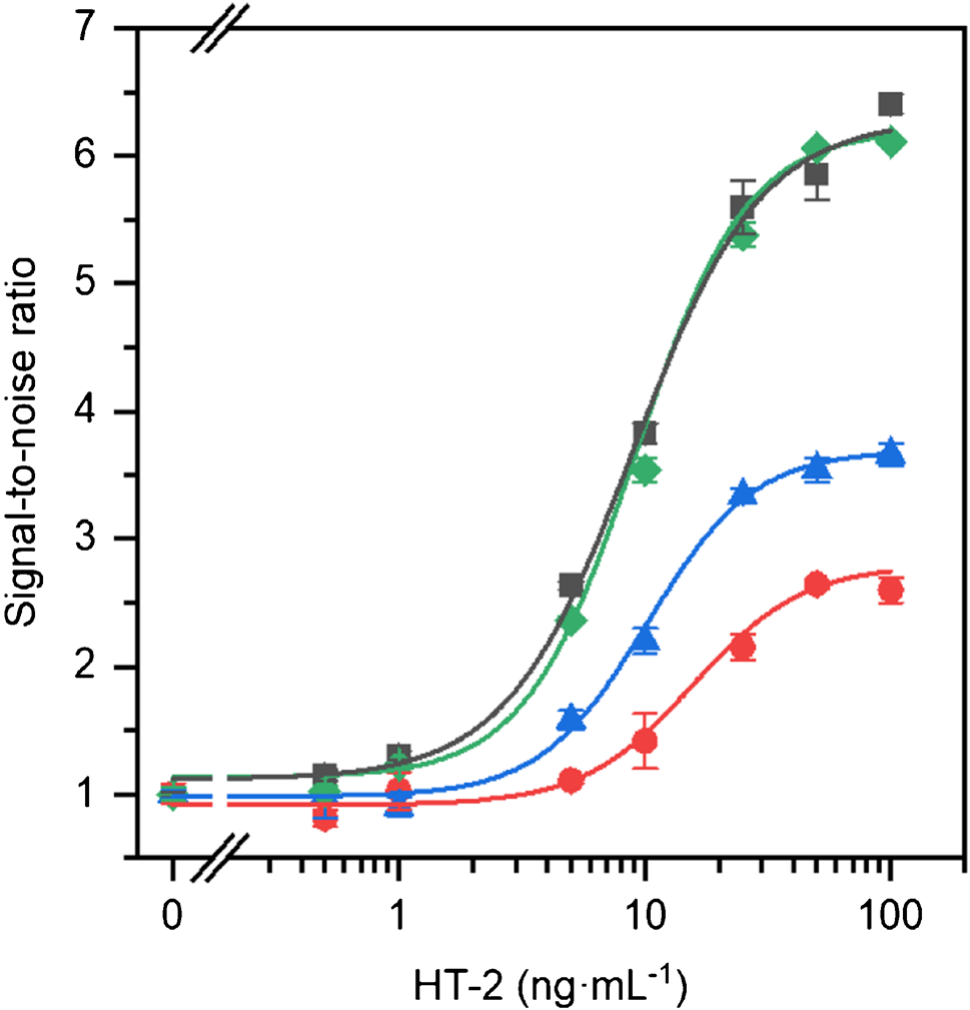
Calibration plots for HT-2 in assay buffer (black squares) and in insect flour extract reconstituted in assay buffer with no dilution (red circles), a 1:1.5 dilution (blue triangles) or a 1:2 dilution (green diamonds). The results are mean signals ± standard errors of the mean (*n* = 3)

Considering the potential impact of biological samples on antibody recognition, we thoroughly evaluated the possibility of a matrix effect using black cricket flour, previously checked to contain none of the HT-2 toxin at quantifiable concentrations [27]. The matrix effects were evaluated by preparing working calibration standards (0–100 ng mL^−^^1^ HT-2) in the blank matrix extracts of the reconstituted black cricket flour, and their analytical signal was compared with that obtained from the standards prepared in the assay buffer. Figure 7 compares the dose–response calibration curves obtained with these standards, revealing no statistically significant differences (95% confidence level) when reconstituted at twice the original volume.

### Method recovery

The extraction procedure’s efficiency was assessed by analyzing enriched samples conducted by spiking black cricket flour blank with HT-2 concentrations ranging from 60 to 200 µg kg^−^^1^, corresponding to 12 to 40 ng mL^−^^1^ concentrations in the undiluted extract. The optimized immunoassay method demonstrates adequate performance for quantifying HT-2 in black cricket flour (Table 1), with recoveries ranging from 91 to 133% and relative standard deviations (RSD) between 6 and 13%. These results indicate that the method is precise and reliable, with efficient recovery of the mycotoxin at different concentrations, making it applicable and highly relevant for analysis in actual black cricket flour samples, demonstrating its practical relevance.

**Table 1:** Optimized immunoassay performance for quantifying HT- 2 mycotoxin in spiked black cricket flour extracts

Nominal concentration (µg·kg^−^^1^)	Estimated concentration (sd) (µg·kg^−^^1^)	Recovery (sd) (%)	RSD (%)
60	79.8 (2.8)	133 (4.7)	6
100	102.1 (7.9)	102 (7.9)	13
200	182.4 (11.2)	91 (5.6)	11

## Conclusions

The immune complex-assay developed in this work enabled the sensitive detection of HT-2 toxin, demonstrating its effective application in analyzing contaminants in food matrices such as cricket flour. With a limit of detection (LOD) of 0.43 ng mL⁻^1^, an EC_50_ of 10.3 ng mL⁻^1^, and a dynamic range of 3.4 to 31 ng mL⁻^1^, this method achieves sensitivity that matches or exceeds existing immunoassays while meeting legislative standards. The assay simplifies procedures compared to enzyme fusion by eliminating the need for substrate addition and prior incubation. It allows for the analysis of up to 96 sample extracts in under 1 h using a microplate reader. The use of recombinant antibodies ensures consistent production and reduces batch variability, while enabling labeling with fluorescent proteins for improved accuracy and decreased optical interferences. Characterized by high sensitivity, selectivity, and reliability, this immunoassay presents a promising method for screening mycotoxins in foods, especially those derived from edible insects. Additionally, computational tools like AlphaFold AI and MOE provided insights into antibody-HT-2 interactions, validating the experimental results.

## Supplementary Information

Below is the link to the electronic supplementary material.

ESM 1 (DOCX 6.81 MB)

ESM 2 (MP4 4.12 MB)

## Data Availability

No datasets were generated or analysed during the current study.
