# Correction: Biotechnology-enhanced immunoassay for accurate determination of HT-2 toxin in edible insect samples

**DOI:** 10.1007/s00604-025-07563-6

**Published:** 2025-09-23

**Authors:** Raúl Sancho-García, Fernando Navarro-Villoslada, Fernando Pradanas-González, Henri O. Arola, Bettina Glahn-Martínez, Tarja K. Nevanen, Elena Benito-Peña

**Affiliations:** 1 Department of Analytical Chemistry, Faculty of Chemistry, Universidad Complutense de Madrid, Plaza de las Ciencias, Ciudad Universitaria, 28040 Madrid, Spain; 2 VTT Technical Research Centre of Finland Ltd, Tekniikantie 21, 02044 Espoo, Finland; 3 Centre for Military Medicine, Finnish Defence Forces, Tukholmankatu 8 A, 00301 Helsinki, Finland

**Correction: Microchimica Acta (2025) 192:301**

10.1007/s00604-025-07146-5

The article "Biotechnology-enhanced immunoassay for accurate determination of HT-2 toxin in edible insect samples", written by Raúl Sancho-García, Fernando Navarro-Villoslada, Fernando Pradanas-González, Henri O. Arola, Bettina Glahn-Martínez, Tarja K. Nevanen & Elena Benito-Peña, was originally published under exclusive license to The Author(s), under exclusive licence to Springer-Verlag GmbH Austria, part of Springer Nature. As a result of the subsequent decision to publish the article under the open access model, the article’s copyright notice was changed on 28 August 2025 to © The Author(s) 2025 and the article is now distributed under a Creative Commons Attribution 4.0 International License, which permits use, sharing, adaptation, distribution and reproduction in any medium or format, as long as you give appropriate credit to the original author(s) and the source, provide a link to the Creative Commons licence, and indicate if changes were made.

The images or other third party material in this article are included in the article’s Creative Commons licence, unless indicated otherwise in a credit line to the material. If material is not included in the article’s Creative Commons licence and your intended use is not permitted by statutory regulation or exceeds the permitted use, you will need to obtain permission directly from the copyright holder.

To view a copy of this licence, visit http://creativecommons.org/licenses/by/4.0.

