# Supplementary material for: Biotechnology-enhanced immunoassay for accurate determination of HT-2 toxin in edible insect samples
Source: Mikrochim Acta. 2025 Apr 16;192(5):301. doi: 10.1007/s00604-025-07146-5 (PMC12433359; doi:10.1007/s00604-025-07146-5)
Supplement: Supplementary file 1 — (DOCX 6.81 MB) [file 604_2025_7146_MOESM1_ESM.docx]

**Biotechnology-Enhanced Immunoassay for Accurate Determination of HT-2 Toxin in Edible Insect Samples**

Raúl Sancho-García,^1,±^ Fernando Navarro-Villoslada,^1,±^ Fernando Pradanas-Gonzalez,^1^ Henri O. Arola,^2^ Bettina Glahn-Martínez,^1*^ Tarja K. Nevanen,^2^ Elena Benito-Peña^1*^

^1^ Department of Analytical Chemistry, Faculty of Chemistry, Universidad Complutense de Madrid, Plaza de las Ciencias, Ciudad Universitaria, 28040 Madrid, Spain

*^2^* *VTT Technical Research Centre of Finland Ltd, Tekniikantie 21, 02044, Espoo, Finland*

^±^ *Equally contributed*

**Contents**

**Anti-IC HT-2 scFv-sfGFP fusion protein preparation 2**

**Table S1 2**

**Figure S1 2**

**Anti-IC HT-2 scFv-sfGFP and anti-HT-2 Fab sequences 3**

**Anti-HT-2 Fab and anti-IC HT-2 scFv-sfGFP prediction structure 3**

**Table S2 4**

**Table S3 5**

**Figure S2 6**

**Figure S3 7**

**Figure S4 8**

**Figure S5 8**

**Figure S6 9**

**Figure S7 10**

**Figure S8 11**

**Table S4 12**

**References 13**

**Anti-IC HT-2 scFv-sfGFP fusion protein preparation**

**Table S1.** Primer sets used for PCR amplification of the pET41 vector containing the sfGFP gene and the anti-IC HT-2 scFv gene fragment. The oligo tail added to the target gene sequence is in boldface, the segment identical with the target gene in italics and the overlapping regions for the Gibson assembly reaction underlined.

| **Vector** | **Primer** | **Sequence 5′→3′** |
| --- | --- | --- |
| Anti-IC HT-2 scFv plasmid | FP | **GGCCATGGCC***CAGGTACAGCTGCAGCAG* |
|  | RP | **AGCTCCTCGCCCTTGCTCAC***GGATCCTCCGCCTGCGG* |
| pET41-sfGFP plasmid | FP | *GTGAGCAAGGGCGAGGAGC* |
|  | RP | **GCTGTACCTG***GGCCATGGCCGGCTGGG* |

**Figure S1.** SDS-PAGE analysis of the purified anti-IC HT-2 scFv-sfGFP fusion protein with Coomassie brilliant blue protein staining: lane 1 and 9, molecular marker (Thermo Scientific™ PageRuler™ Prestained Protein Ladder, 10 to 180 kDa); 2, lysate sample; 3, HisTrap column throughput with non-retained proteins; 4-7, aliquots from the HisTrap column elution; 8, purified protein obtained after PD-10 column. Prior to analysis, a 5-minute boiling step at +95 °C was used. Molecular weight of the fusion protein: 56.6 kDa.

**Anti-IC HT-2 scFv-sfGFP and anti-HT-2 Fab sequences**

- **(scFv-VH)-(Linker)-(scFv-VL)-(Linker)-(GFP)-(HisTag)**

QVQLQQSGAEVKKPGASVKVSCKASGYTFT***SYYMH***WVRQAPGQGLEWMG***IINPSGGSTSYAQKFQ***GRVTMTRDTSTSTVYMELSSLRSEDTAVYYCAR***DEGYGDYVY***WGQGTLVTVSSLEGGGGSGGGGSGGGGSELQAVLTQPSSVSGAPGQRVTISC***TGSSSNIGAGYDVH***WYQQLPGTAPKLLIY***GNNNRPS***GVPDRFSGSKSGTSASLAISGLQSEDEADYYC***ATWDDSLNGVV***FGGGTKVTVLGAAAGGGSVSKGEELFTGVVPILVELDGDVNGHKFSVRGEGEGDATNGKLTLKFICTTGKLPVPWPTLVTTLTYGVQCFSRYPDHMKRHDFFKSAMPEGYVQERTISFKDDGTYKTRAEVKFEGDTLVNRIELKGIDFKEDGNILGHKLEYNFNSHNVYITADKQKNGIKANFKIRHNVEDGSVQLADHYQQNTPIGDGPVLLPDNHYLSTQSVLSKDPNEKRDHMVLLEFVTAAGITHGMDELYKHHHHHHHH

***CDR 1* (H: 31 – 35, L: 23 – 36)**

***CDR 2* (H: 50 – 65, L: 52 – 58)**

***CDR 3* (H: 99 – 107, L: 91 – 101)**

- **(Fab-HC):(Fab-LC)**

DVQLQESGPGLVKPSQSLSLTCSVTGYSIT***SGYFWN***WIRQFPGNKLEWMG***YIRYDGNKDYNPSLKN***RISITRDTSKNQFFLKLNSVTTEDTATYYCAR***VRYDVNY***WGPGTSVTVSSAKTTPPSVYPLAPGSAAQTNSMVTLGCLVKGYFPEPVTVTWNSGSLSSGVHTFPAVLQSDLYTLSSSVTVPSSTWPSETVTCNVAHPASSTKVDKKIVPRDCAAAHHHHHH**:**DIVMTQSQKFMSTSVGDRVTITC***KASQNVRSAVA***WYQQKPGQSPKALIY***SASYRYS***GVPNRFTGGGSGTDFTLTISNVQSEDLAEYFC***QQYNSYPLT***FGSGTKLDLKRADAAPTVSIFPPSSEQLTSGGASVVCFLNNFYPKDINVKWKIDGSERQNGVLNSWTDQDSKDSTYSMSSTLTLTKDEYERHNSYTCEATHKTSTSPIVKSFNRNEC

***CDR 1* (H: 31 – 36, L: 24 – 34)**

***CDR 2* (H: 51 – 66, L: 50 – 56)**

***CDR 3* (H: 99 – 105, L: 89 – 97)**

**Anti-HT-2 Fab and anti-IC HT-2 scFv-sfGFP prediction structure**

The fragment antigen-binding region (anti-HT-2 Fab) structure was predicted by the AlphaFold2 model (AF2) using the Neurosnap with the ColabFold implementation [1]. In contrast, the anti-HT-2 single-chain fragment variable linked with a green fluorescence protein (anti-IC HT-2 scFv-sfGFP) was predicted by the AlphaFold3 model (AF3) using the Google DeepMind platforms respectively [2]. The parameters used for the AF2 and AF3 prediction of the anti-HT-2 Fab and anti-IC HT-2 scFv-sfGFP, respectively, are shown in **Table S2**. By default, both platforms produce five structures. The accuracy of the predicted structures was assessed by four confidence metrics generated by both models, including the average predicted local distance difference test (pLDDT), the maximum predicted aligned error (PAE), and the predicted template modeling (pTM) (**Table S3**). The pLDDT score averaged across all residues designates the overall confidence for the whole protein chain. The pLDDT score ranges between 0 and 100. A high pLDDT (e.g., > 80) indicates high confidence in its prediction, whereas a low pLDDT (e.g., < 50) means that the predicted structure is not very confident and may suggest that the residues are in intrinsically disordered protein regions. Furthermore, high pLDDT scores in some areas of a protein mean that the AF2 model is confident about the positions of those residues. The pLDDT scores and their structures for the five anti-HT-2 Fab and anti-IC HT-2 scFv-sfGFP predicted structures are shown in **Figure S2** and **Figure S3**, respectively. The PAE score indicates the confidence level of each amino acid residue pair, i.e., the expected positional error or variance in angstroms at some residue (scored residue) when the predicted and true structures are aligned on another residue (aligned residue). PAE values range between 0 and 30 per position, where zero means the model is very confident in the residue-residue interactions, and 30 means low confidence in the residue-residue interactions—residues within the same domain exhibit lower PAEs than the inter-domain residues. The PAE scores for the five anti-HT-2 Fab-predicted and anti-IC HT-2 scFv-sfGFP-predicted structures are shown in **Figure S4** and **Figure S5**, respectively. The pTM score is a metric derived from the template modeling score (TM-score) measure that measures the accuracy of the protein's overall structure by comparing the predicted and the hypothetical true structure [3]. pTM values range between 0 and 1. Higher pTM values tend to be better predictions. A pTM score greater than 0.75 can be interpreted as a reasonable prediction.

**Table S2**. Parameters used for AF2 and AF3 models to predict the anti-HT-2 Fab and anti-IC HT-2 scFv-sfGFP structure.

| **Parameter** | **anti-HT-2 Fab**  **(AF2 model)** | **anti-IC HT-2 scFv-sfGFP (AF3 model)** |
| --- | --- | --- |
| Custom MSA | None | None |
| Custom Template | None | None |
| MSA Mode | MMseqs2 (UniRef+Environmental) | - |
| Model Type | alphafold2_multimer-v3 | - |
| Number Ensembles | 1 | - |
| Number Recycles | 6 | - |
| Pair mode | unpaired + paired |  |
| Recycles Erly Stop Tolerance | 0.0 | - |
| Template Mode | pdb70 | - |
| Training Mode | No | - |
| Amber relaxation | No | - |
| Seeds | - | 1000 |

**Table S3**. Confidence metrics from the AF2 and AF3 models for the anti-HT-2 Fab and anti-IC HT-2 scFv-sfGFP structure prediction, respectively.*^a^*

| **Rank** | **Mean pLDDT***^b^* | | **Maximum PAE***^c^* | | **pTM***^d^* | |
| --- | --- | --- | --- | --- | --- | --- |
|  | **anti-HT-2 Fab** | **anti-IC HT-2 scFv-sfGFP** | **anti-HT-2 Fab** | **anti-IC HT-2 scFv-sfGFP** | **anti-HT-2 Fab** | **anti-IC HT-2 scFv-sfGFP** |
| 1 | 94.37 | 86.08 | 31.41 | 31.71 | 0.88 | 0.53 |
| 2 | 94.22 | 85.76 | 31.25 | 31.66 | 0.86 | 0.53 |
| 3 | 93.92 | 85.93 | 31.33 | 31.61 | 0.85 | 0.53 |
| 4 | 93.70 | 85.90 | 31.30 | 31.60 | 0.85 | 0.52 |
| 5 | 94.01 | 85.81 | 31.36 | 31.60 | 0.85 | 0.51 |

*^a^*anti-HT-2 Fab: residue position. Anti-IC HT-2 scFv-sfGFP: atomic position.

*^b^*pLDDT: predicted Local Distance Difference Test.

*^c^*PAE: Predicted Aligned Error.

*^d^*pTM: predicted Template Modeling.

**(A)**

**
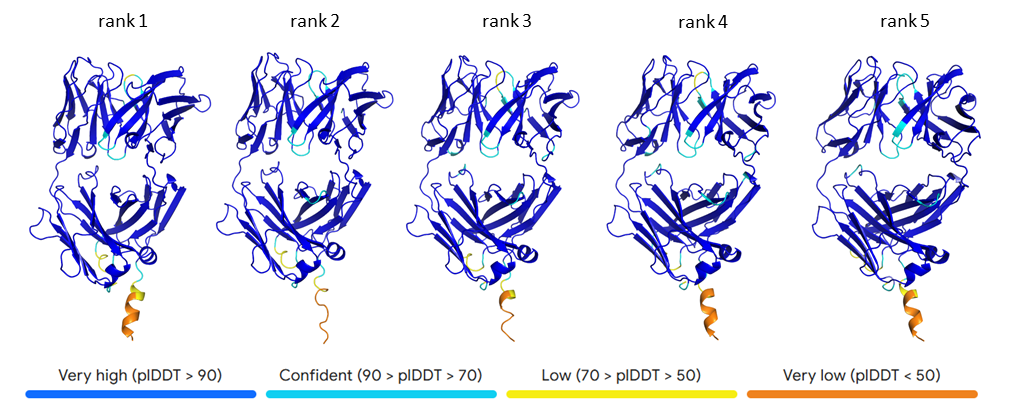
(B)**

**Figure S2**. **(A)** AF2 predicted Local Distance Difference Test (pLDDT) by residue position and **(B)** predicted molecular structures for the anti-HT-2 Fab.

**(A)**


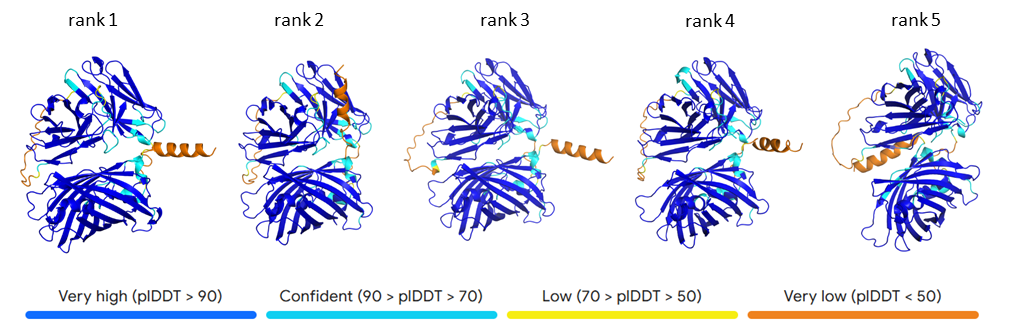
**(B)**

**Figure S3**. **(A)** AF3 predicted Local Distance Difference Test (pLDDT) by atom position and **(B)** predicted molecular structures for the anti-IC HT-2 scFv-sfGFP.

**Figure S4**. Predicted Aligned Error (PAE) map for the anti-HT-2 Fab predicted structures.

**Figure S5**. Predicted Aligned Error (PAE) map for the anti-IC HT-2 scFv-sfGFP predicted structures.

**
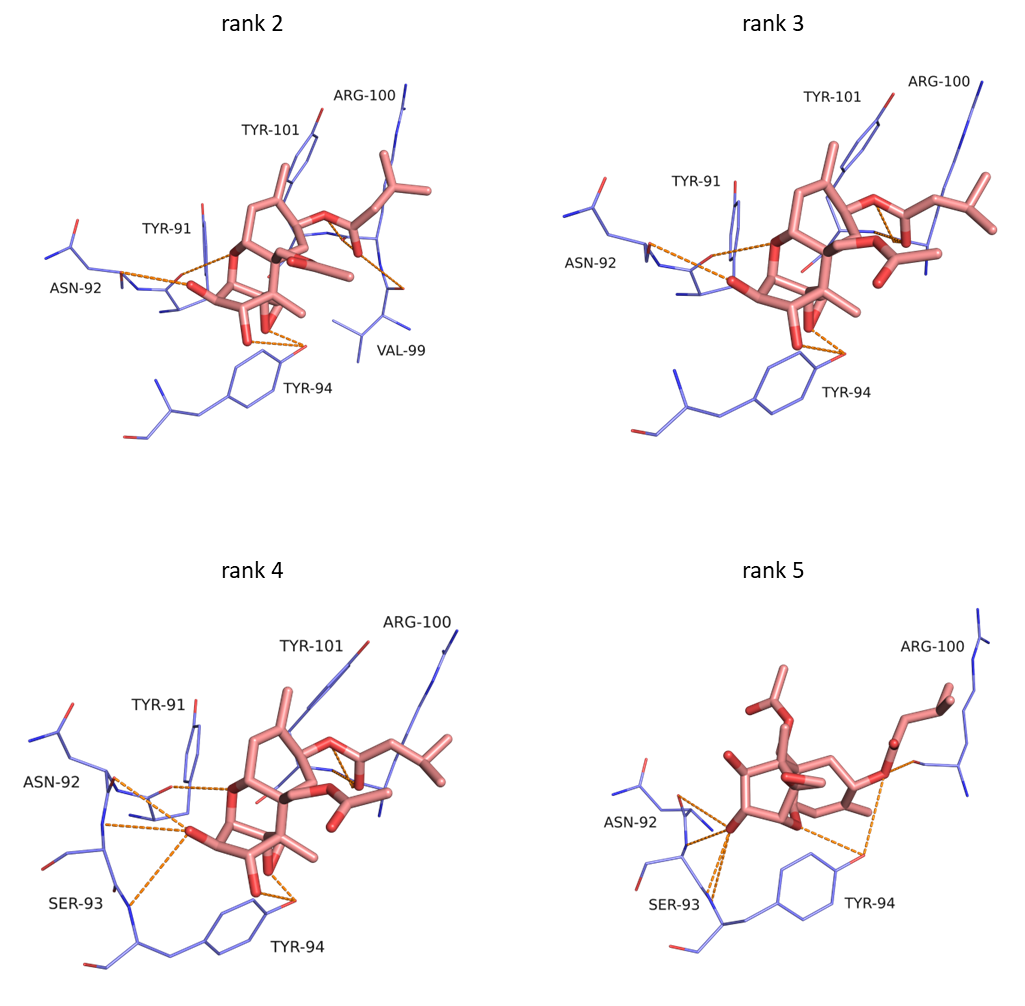
**

**Figure S6**. Four next top-ranked MOE predictions of the anti-HT-2 Fab interaction against HT-2 mycotoxin.


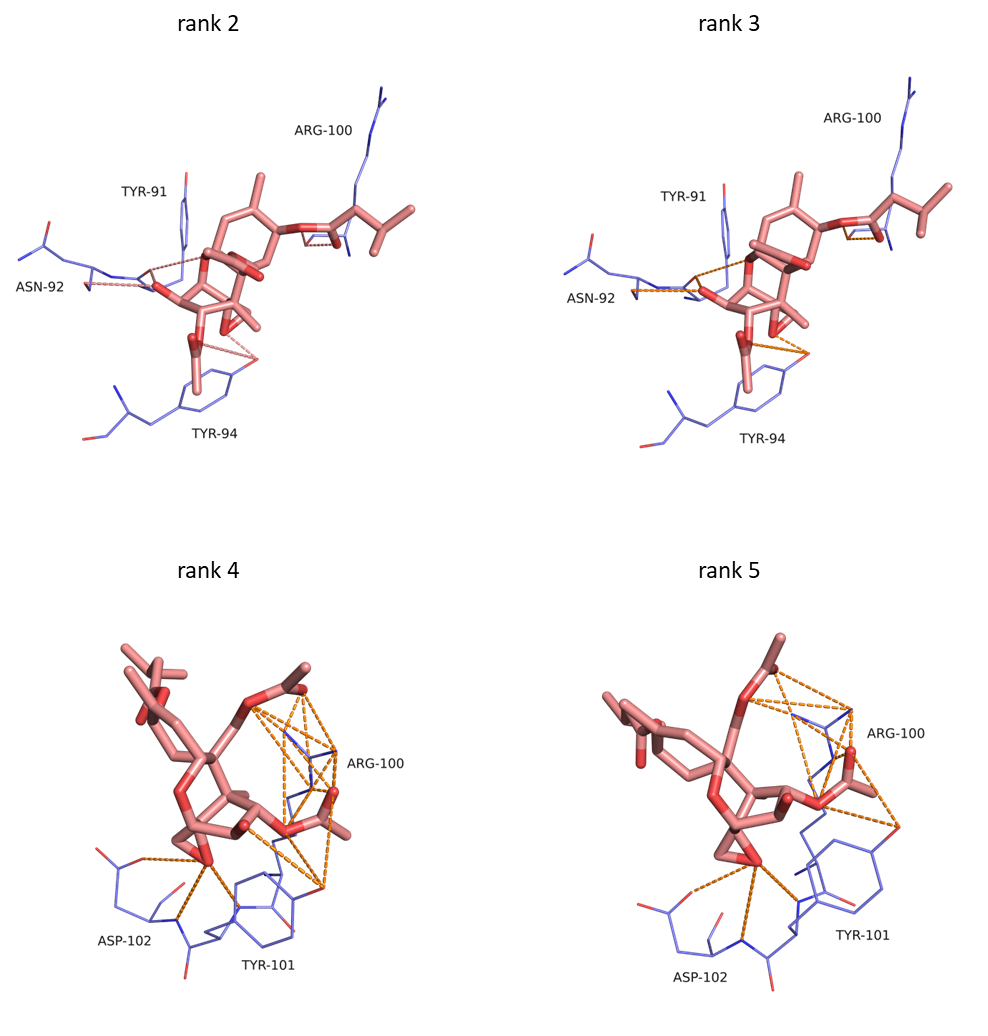


**Figure S7**. Four next top-ranked MOE predictions of the anti-HT-2 Fab interaction against T-2 mycotoxin.


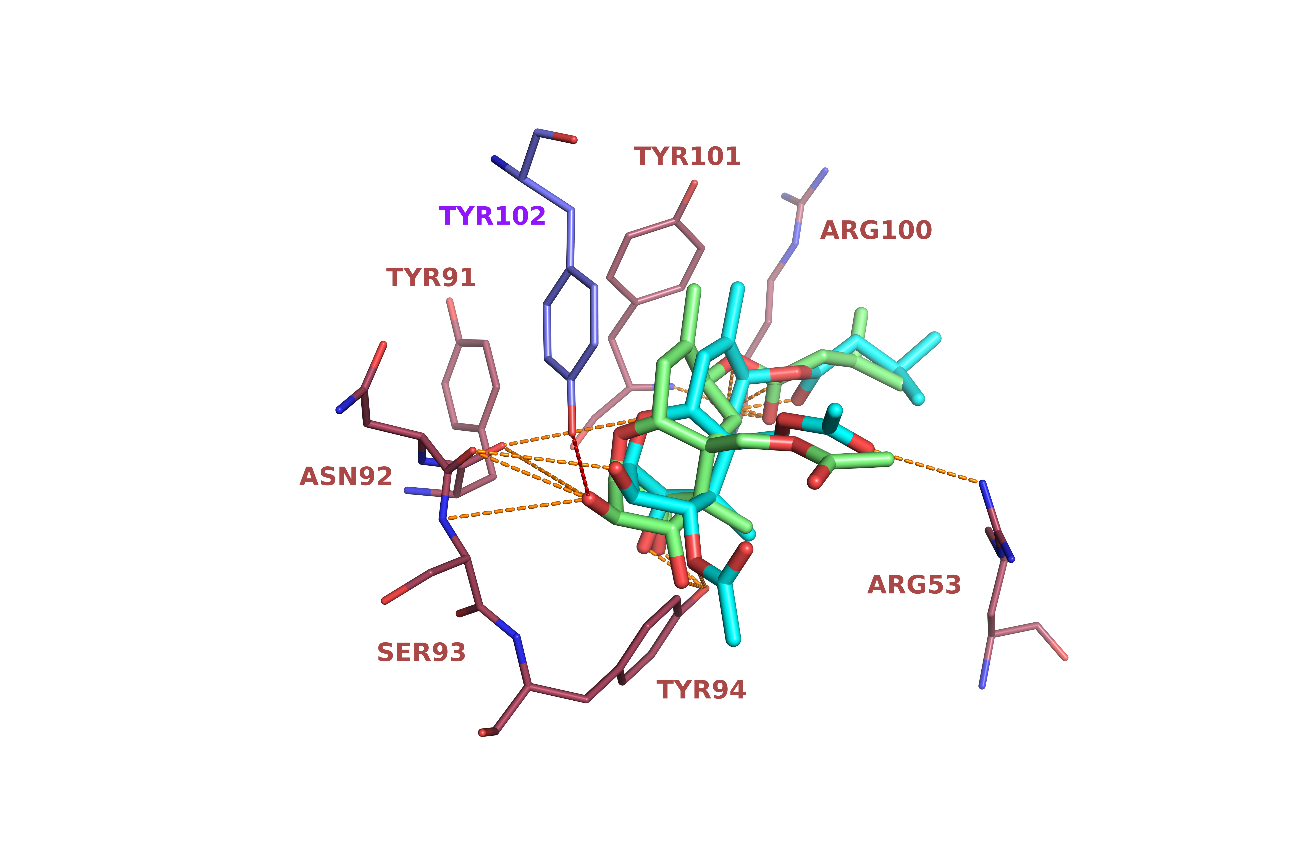


**Figure S8**. Top ranked MOE prediction of the anti-HT-2 Fab/mycotoxin complex interaction against anti-IC HT-2 scFv-sfGFP for HT-2 (green) and T-2 (cyan) toxins.

**Table S4.** Comparative summary of analytical methods reported for the analysis of HT-2 in different samples.

|  | **Sample** | **Method** | **Sample treatment** | **LOD/LOQ** | **Dynamic range** | **Ref.** |
| --- | --- | --- | --- | --- | --- | --- |
| Chromatographic | Barley  Maize  Rice  Wheat | UHPLC-MS/MS | 5% formic acid in acetonitrile (QuEChERS) evaporation to dryness under N_2_ stream | 1.3/4.2 μg·kg^-1^  1.4/4.8 μg·kg^-1^  1.4/4.5 μg·kg^-1^  1.5/4.8 μg·kg^-1^ | 4.2-500 μg·kg^-1^  4.8-500 μg·kg^-1^  4.5-500 μg·kg^-1^  4.8-500 μg·kg^-1^ | [4] |
|  | Edible insect | UHPLC-MS/MS | Extraction with NADES: choline chloride/urea (mole ratio 1:2) with 15% water, filtration and 1:1 dilution water | 80/260 | 260 – 1500 | [5] |
|  | Bee pollen, propolis, honey, and royal jelly | HPLC-UV//LC-MS/MS | Methanol extraction, filtration evaporation and dilution acetonitrile:water (21:4 v/v) | 0.002 ng·mL^-1^ | 125 – 1000 ng·mL^-1^ | [6] |
|  | Yellow mealworm | LC-MS/MS | 0.2% formic acid/acetonitrile (50/50, v/v) extraction and 1:10 dilution with water | 8.4/27.9 μg·kg^-1^ |  | [7] |
|  | Cereals | UPLC/FLD | Methanol–water (90–10, *v*/*v*) extraction, filtration, 1:5 dilution with water, filtration, immunoaffinity column, evaporation and derivatization | -/19 μg·kg^−1^ | - | [8] |
| Immunoassays |  | ELISA | Extraction buffer | 75* μg kg^−1^ | - |  |
|  | Wheat | FPIA | Methanol/water (90/10, *v*/*v*) extraction, filtration and 1:5 (*v*/*v*) dilution with 4% NaCl solution | 10 µg·kg^-1^ | 0.1 – 73.2* ng·mL^-1^ | [9] |
|  | Wheat | TR-FRET | Methanol:water (70:30) extraction, filtration and 1:10 dilution with PBS | 0.38 ng·mL^-1^  (19 μg·kg^-1^) | 0.5 – 8 ng·mL^-1^  (25 – 400 µg·kg^-1^) | [10] |
|  | Barley | LFIA | 80% (v/v) methanol/water extraction, 12.5:87.5 dilution with Tris buffer | - | 80** µg·kg^-1^ | [11] |
|  | FBS | DPV | Dilution with buffer in 1:1 ratio | 1.6 ng∙mL^-1^ | 6.3 – 100.0 ng∙mL^-1^ | [12] |
|  | Black cricket flour | IC-FIA | Methanol extraction | 0.43 ng∙mL^-1^  (2.12 μg·kg^-1^) | 3.4 – 31 ng∙mL^-1^  (17 – 155 μg·kg^-1^) | This work |

DPV: differential pulse voltammetry; FBS: Fetal bovine serum; FIA: fluorescence immunoassay; FLD: fluorescence detection; FPIA: fluorescence polarization immunoassay; HPLC: High Performance Liquid Chromatography; IC: immunocomplex; LC: Liquid Chromatography; LFIA: lateral flow immunoassay; LOD: limit of detection; LOQ: limit of quantification; MS/MS: tandem mass spectrometry; NADES: Natural deep eutectic solvent; TR-FRET: Time-resolved fluorescence energy transfer; UHPLC: Ultra High Performance Liquid Chromatography; UPLC: Ultra Performance liquid chromatography.

* Expressed as the sum of the T-2 and HT-2 toxins. ** Cutoff value

**References**

1. Neurosnap - Computational Biology, Simplified. https://neurosnap.ai/. Accessed 30 Nov 2024

2. AlphaFold Server. https://alphafoldserver.com/. Accessed 30 Nov 2024

3. Zhang Y, Skolnick J (2004) Scoring function for automated assessment of protein structure template quality. Proteins Struct Funct Bioinf 57:702–710. https://doi.org/10.1002/prot.20264

4. Mahdjoubi CK, Arroyo-Manzanares N, Hamini-Kadar N, et al (2020) Multi-Mycotoxin Occurrence and Exposure Assessment Approach in Foodstuffs from Algeria. Toxins 12:194. https://doi.org/10.3390/toxins12030194

5. Pradanas-González F, Álvarez-Rivera G, Benito-Peña E, et al (2021) Mycotoxin extraction from edible insects with natural deep eutectic solvents: a green alternative to conventional methods. J Chromatogr A 1648:462180. https://doi.org/10.1016/j.chroma.2021.462180

6. Keskin E, Eyupoglu OE (2023) Determination of mycotoxins by HPLC, LC-MS/MS and health risk assessment of the mycotoxins in bee products of Turkey. Food Chemistry 400:134086. https://doi.org/10.1016/j.foodchem.2022.134086

7. Piacenza N, Kaltner F, Maul R, et al (2021) Distribution of T-2 toxin and HT-2 toxin during experimental feeding of yellow mealworm (Tenebrio molitor). Mycotoxin Res 37:11–21. https://doi.org/10.1007/s12550-020-00411-x

8. D’Agnello P, Vita V, Franchino C, et al (2021) ELISA and UPLC/FLD as Screening and Confirmatory Techniques for T-2/HT-2 Mycotoxin Determination in Cereals. Applied Sciences 11:1688. https://doi.org/10.3390/app11041688

9. Lippolis V, Porricelli ACR, Mancini E, et al (2019) Fluorescence Polarization Immunoassay for the Determination of T-2 and HT-2 Toxins and Their Glucosides in Wheat. Toxins 11:380. https://doi.org/10.3390/toxins11070380

10. Arola HO, Tullila A, Kiljunen H, et al (2016) Specific Noncompetitive Immunoassay for HT-2 Mycotoxin Detection. Anal Chem 88:2446–2452. https://doi.org/10.1021/acs.analchem.5b04591

11. Foubert A, Beloglazova NV, Gordienko A, et al (2017) Development of a Rainbow Lateral Flow Immunoassay for the Simultaneous Detection of Four Mycotoxins. J Agric Food Chem 65:7121–7130. https://doi.org/10.1021/acs.jafc.6b04157

12. Kudr J, Zhao L, Nguyen EP, et al (2020) Inkjet-printed electrochemically reduced graphene oxide microelectrode as a platform for HT-2 mycotoxin immunoenzymatic biosensing. Biosensors and Bioelectronics 156:112109. https://doi.org/10.1016/j.bios.2020.112109
